# Explaining factors influencing students' depression with a deep learning approach

**DOI:** 10.3389/fpsyg.2025.1684274

**Published:** 2026-01-29

**Authors:** Xinyu Li, Yunyi Hu, Huohong Chen, Xingxing Wang, Feng Gong

**Affiliations:** 1 School of Computer Science and Engineering, Central South University, Changsha, China; 2 School of Information Resource Management, Renmin University of China, Beijing, China; 3 School of Educational Science, Hunan Normal University, Changsha, China; 4 School of Marxism, Central South University, Changsha, China

**Keywords:** contribution analysis, deep learning, depression, high education, Mamba, mental health

## Abstract

**Objective:**

Student mental health has emerged as an increasingly prominent issue in sustainable educational healthcare systems. Accurately and promptly identifying students' depression and analyzing the key factors associated with it are crucial for improving student mental health.

**Method:**

We propose an artificial intelligence algorithm, GLNet, that integrates Mamba and convolutional layers to extract features from students' demographic, academic, and lifestyle information for depression analysis. The performance of GLNet is validated on the publicly available Student Depression Dataset.

**Results:**

GLNet achieves an accuracy of 88.84% on the Student Depression Dataset, outperforming other methods and verifying its effectiveness in student depression analysis. Factor contribution analysis indicates that academic pressure and financial stress may be associated with student depression, while healthy dietary habits and academic satisfaction may alleviate depression. Subgroup analysis further reveals that a higher Cumulative Grade Point Average may be positively correlated with depression in female students, and unhealthy dietary habits may be linked to depression among doctoral students.

**Conclusion:**

GLNet can serve as a reliable tool for enhancing student mental health. It also provides valuable insights for educators to identify students at risk of depression, contributing to the optimization of student mental health intervention strategies.

## 1 Introduction

Depression is a common mental illness characterized by persistent low mood, loss of interest or pleasure in activities, impaired memory, and feelings of fatigue or lack of energy (Arnone et al., 2024; Li et al., 2025; Maes et al., 2024; Haghish and Czajkowski, 2024). With the rapid development of society and the increasing pace of life, the incidence of depression among student populations has shown an upward trend (Liu et al., 2022). According to the World Health Organization (WHO), there are over 400 million people suffering from depression worldwide (Liu et al., 2024), posing a huge challenge to public health systems (Yılmaz et al., 2025). Depression can lead to a decline in students' academic performance and social abilities, and trigger a chain reaction, such as communication barriers between students and teachers, which severely hinders students' overall development (Verboom et al., 2014; Sörberg Wallin et al., 2019). Therefore, designing an accurate depression diagnosis mechanism for students and quantifying the risk factors is a necessary measure to improve students' mental health.

The etiology of depression can be understood through a biopsychosocial model, which posits that depression arises from a complex interplay of biological vulnerabilities, psychological processes, and social-contextual factors (Borrell-Carrió et al., 2004; Schotte et al., 2006; Maxwell and Cole, 2009). Several theoretical models elaborate on the multifaceted mechanisms underlying student depression. Specifically, research related to the interaction mechanism of social structure, stress, and mental health (Aneshensel et al., 1991; Morris et al., 2008), including the Diathesis-Stress Model (Colodro-Conde et al., 2018; Arnau-Soler et al., 2019; Harkness and Stewart, 2026), posits that external environmental stressors, such as academic pressure, financial difficulties, and interpersonal conflicts, interact with individuals' inherent vulnerabilities, thereby increasing the risk of depression. Beck's Cognitive Theory of Depression (Haaga et al., 1991; Haaga and Beck, 1995; Zaiden and Mahfar, 2024) emphasizes that hopelessness about the future may be associated with negative cognitive biases, including selective attention to adverse information, overgeneralization, and catastrophizing thinking, which in turn predispose individuals to depression. Social Role Theory (Kim and Kwon, 2025; Hsu et al., 2021) explains gender differences in depression risk, attributing these disparities to distinct social roles and expectations assigned to men and women. Specifically, women often face dual role conflict, as they are pressured to achieve academic excellence while conforming to traditional feminine role norms, inducing psychological stress. Therefore, these models reveal how external stressors, cognitive processing biases, and social structural factors interact to shape students' depression risk, highlighting the “external-internal interaction” nature of depressive pathogenesis.

Research on the mechanisms underlying the interactions among psychological needs, development, and mental health (Tang et al., 2020), such as Self-Determination Theory (Moore et al., 2021; Leow et al., 2016), identifies three basic psychological needs, including autonomy, competence, and relatedness. Satisfaction of these needs enhances intrinsic motivation and promotes mental health, whereas unmet needs may induce depression. For example, students' satisfaction with their learning experience may serve as a key factor influencing their risk of depression. Borbély's Two-Process Model (Borbély, 1987; Abhilash and Shafer, 2024) explains how sleep demand accumulates with wakefulness duration, as insufficient sleep impairs individuals' emotional regulation, thereby increasing the risk of depression. Additionally, Erikson's Theory of Psychosocial Development (Knight, 2017; Degges-White, 2017; Sekowski et al., 2024) reveals that individuals face unique developmental tasks and psychological challenges at different stages of life. As individuals age, their cognitive abilities, such as rational analysis and emotional regulation, gradually improve, and psychological resilience is enhanced. However, the dominant risk factors for depression vary across developmental stages, implying potential differences in the manifestations of depression among students of different educational levels and age groups. These models supplement understanding of depression mechanisms by focusing on individuals' internal psychological needs, developmental laws, and basic physiological regulation, emphasizing that the fulfillment of intrinsic needs and the maintenance of developmental balance are crucial for analyzing student depression.

Traditional statistical methods and many early machine learning models often assess them in isolation. These approaches fail to capture the complex, non-linear interplay between factors, which is central to the psychopathology of depression. Artificial intelligence algorithms have been proven capable of modeling the relationship between factors and outcomes, and have shown effectiveness in the diagnosis of individuals' mental health (Mimikou et al., 2025; Zhang X. et al., 2023; Algarni and Thayananthan, 2025). Specifically, Hong et al. (2025) used logistic regression to model factors in disabled elderly individuals to predict their levels of depression. Vu et al. (2025) employed XGBoost to model individual information such as academic, and financial status to predict depression. Risqi et al. (2025) used SVM to model behavioral data of college students to predict whether they have persistent depression. Gohari et al. (2024) used random forests to model factors such as family life and school belongingness in Canadian adolescents to predict depression. Song et al. (2025) used LightGBM to model factors in elderly individuals to predict their depression. However, these methods struggle to explore the deeper information in the data and are highly dependent on manually selected high-quality features.

Deep learning can extract deep information from the data through multiple hidden layers, enabling the exploration of complex patterns in factors for depression (Zamani et al., 2025). Specifically, Baek and Chung (2020) used multiple separate dense layers to model the lifestyle and health status of individuals from the Korean National Health and Nutrition Examination Survey to predict their depression levels. Saha et al. (2024) proposed DeprMVM, which uses a multilayer perceptron (MLP) to extract deep features from individuals' psychological and sociodemographic information, followed by using SVM for depression prediction. However, these methods struggle to model the global contextual relationships between factors, limiting the performance of the model. Additionally, Mamba (Dao and Gu, 2024) has shown potential in computer vision and text processing by using structured spatial models (SSM) to efficiently extract global information from input data and improve the linear scalability of data processing. However, Mamba's potential in enhancing depression detection in students has not been fully explored. Global information can reveal trends in the data and the relationships between distant factors (Tang et al., 2024; Khan and Ghosh, 2021). Local information can capture the details within the data (El-Rashidy et al., 2024; Kostopoulos et al., 2019). Exploring and combining both global and local information in the data could provide a more comprehensive perspective, helping to further improve the performance of student depression prediction.

Explaining the decision-making process of deep learning methods may help educators better identify the triggers of depression. However, deep learning methods are often regarded as a black-box approach, making their interpretability analysis a challenge (Xu and Yang, 2025). The Shapley Additive Explanations (SHAP) method (Lundberg and Lee, 2017), as a game-theory-based interpretability technique, provides a powerful tool for the interpretability of deep learning models. SHAP quantifies the importance of features by calculating their marginal contributions to the model's output and reveals the decision-making rationale of the model in a consistent and interpretable way. Therefore, using the SHAP method to explain the impact of different factors on student depression may be effective for educators in enhancing students' mental health.

To address this gap, we propose GLNet, a deep learning model designed to concurrently analyze both local and global features within a student's profile. Crucially, by integrating GLNet with SHAP, an explainability method, we aim not only to predict but to explain the drivers of depression, turning a “black box” into a transparent tool for psychological insight. Specifically, this study aims to: (1) Validate the performance of GLNet in accurately identifying student depression. (2) Identify and quantify the key risk and protective factors and their complex contributions. (3) Investigate the heterogeneity of these factors across different student subgroups, providing tailored insights for targeted interventions.

## 2 Materials and methods

### 2.1 Dataset description

We use the publicly available Student Depression Dataset (SDD; Jurado, 2025; Shamim, 2025). SDD was originally gathered through anonymized, self-reported surveys distributed across various educational institutions, and sensitive information was removed or encoded to protect individual privacy, ensuring compliance with ethical research standards. This dataset contains 27,901 student samples. After removing samples with missing information, a total of 27,898 samples are used in this study. Each student sample includes their demographic information, academic indicators, lifestyle, wellbeing, other additional factors, and whether they are experiencing depression using standardized depression scales. We follow data processing approaches (Zeng et al., 2025; Chowdhury and Das, 2025; Raza et al., 2025; Risqi et al., 2025) for SDD, ensuring that all student information is fully anonymized without any disclosure of personal privacy information. Moreover, demographic factors include the age of the student in years, the gender of the student, and the city or region where the student resides. Academic indicators include the cumulative grade point average of the student, how satisfied the student is with their studies, and the level of pressure the student faces in academic settings, such as stress from exams, assignments, and overall academic expectations. Lifestyle and Wellbeing factors include the average number of hours the student sleeps per day, the student's eating patterns and nutritional habits, the average number of hours per day the student dedicates to study, the student's satisfaction with their work environment, and the pressure related to work responsibilities. Additional factors include the field of study of the student, the academic degree or program that the student is pursuing, whether the student has ever experienced suicidal ideation, whether the student has a family history of mental illness, and the degree of stress experienced due to financial concerns.

We split the dataset into training and testing sets with an 80:20 ratio, with 15% of the training set used as a validation set for hyperparameter tuning. Table 1 shows the demographic characteristics of the data. Notably, the depression status, gender, and age of the students in the training and testing dataset remain consistent.

**Table 1:** Summary of the datasets used in this study.

**Demographic**	**Training data**	**Testing data**
Subject, *n*	22,318	5,580
Male, *n*	12,396 (55%)	3,150 (55%)
Age, mean	22.8	22.8
**Mental health**, ***n***		
Non-depression	9,250 (41%)	2,313 (41%)
Depression	13,068 (59%)	3,267 (59%)

### 2.2 Data encoding

To analyze students' depression using the artificial intelligence algorithm GLNet, we have performed one-hot encoding on students' potential depression risk factors and their depression status. Specifically, students with a depressed status are encoded as 1, and those with a non-depressed status are encoded as 0. To facilitate the factor contribution analysis and quantify the impact of different factors on student depression, we have re-encoded the factors “Profession,” “Degree,” “City,” “Sleep Duration” and “Dietary Habits.” Specifically, we have split these factors into multiple binary factors. Finally, we obtain 114 encoded factors as inputs for GLNet.

### 2.3 Model development

The overall pipeline of our model can be divided into three steps: (1) We use global and local blocks (GLBs) to extract global and local features to predict students' depression. (2) We input the prediction results of GLNet into a weighted cross-entropy loss function to optimize the trainable parameters within GLNet. (3) We use SHAP to quantify the contribution of factors in the trained GLNet model's decision-making process, thereby explaining the impact of different factors on students' depression.

#### 2.3.1 Structure of GLNet

To facilitate the subsequent extraction of global and local features, we first use a fully connected layer to linearly map the encoded 114 student features to 128 deep features, which can be expressed as:


$$\begin{array}{c} x_{fc}=f_{fc}\left(x\right), \end{array}$$ (1)

where *f*_*fc*_ is the fully connected layer. We then input the *x*_*fc*_ into GLBs to explore the global and local features in the data, and employ residual connections to aggregate the shallow and deep features of the network for student depression prediction.

**Figure 1:**
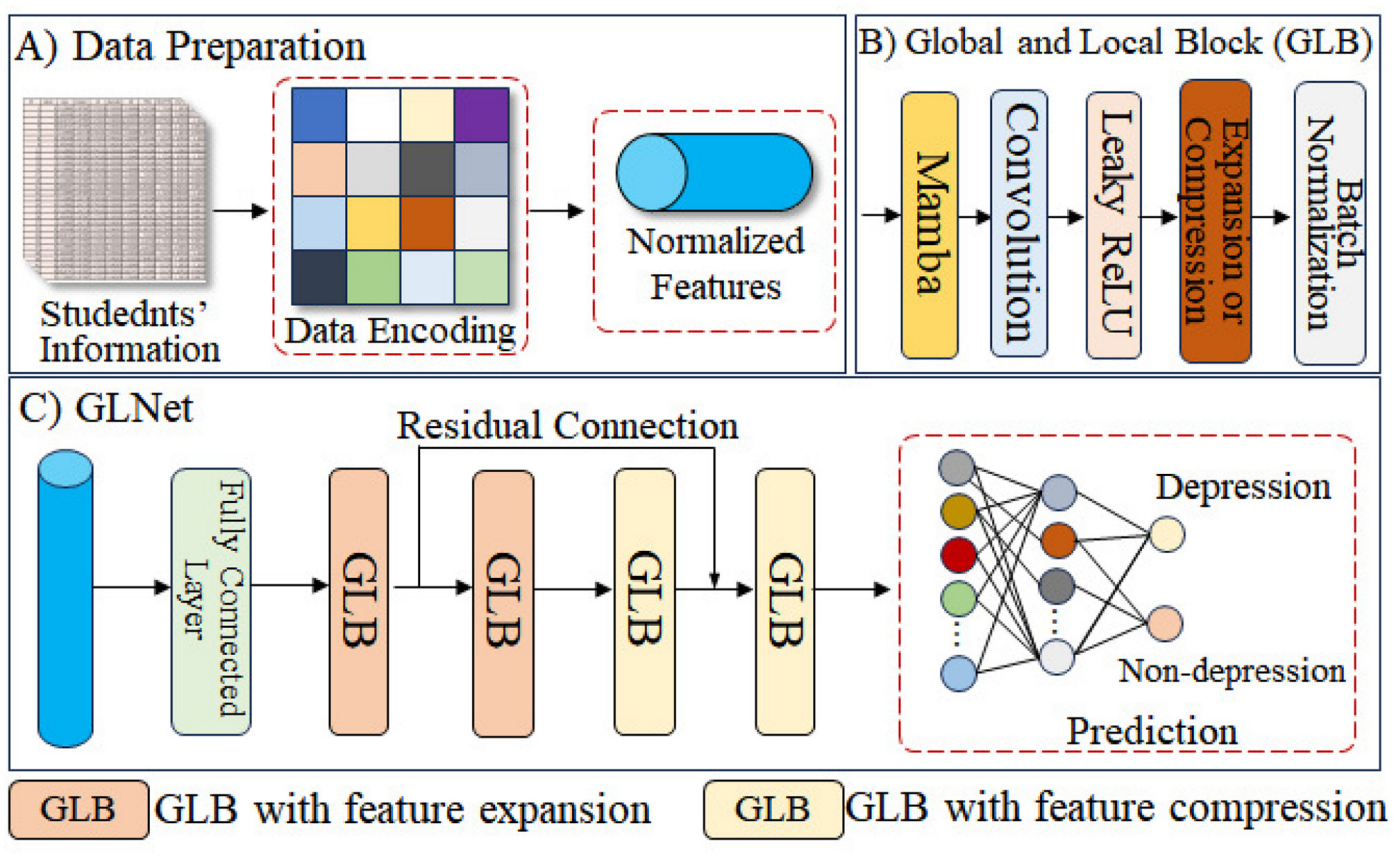
Overall pipeline of the proposed GLNet. **(A)** All data are encoded and normalized to be input into the network. **(B)** The Global and Local Block is employed for deep feature extraction. **(C)** GLNet performs student depression analysis.

Global and local block for feature extraction: as shown in Figures 1B, C, GLNet is primarily composed of GLBs. The GLBs use Mamba and convolutional layers, respectively, to explore the global and local features in students' academic and lifestyle information. Specifically, the Mamba layer (Dao and Gu, 2024), based on State-Space Models (SSM), introduces dynamic systems that can adaptively update based on the current state of the sequence, which allows for more efficient modeling of long-range dependencies in the input data. The Mamba layer can be represented as:


$$\begin{array}{c} f_{mamba}\left(h_{t}\right)=F\left(h_{t-1},x_{t},\theta \right), \end{array}$$ (2)

where *h*_*t*_ is the state at time step *t*. *x*_*t*_ is the input at time step *t*. θ represents the parameters of the model, and F represents the function that updates the state at each time step based on the previous state and the current input. Additionally, Convolution is regarded as an effective method for extracting local information from data due to its localized receptive field (Sadasivan et al., 2025; Kim et al., 2025). Therefore, the process of GLBs can be represented as:


$$\begin{array}{c} f_{glb}\left(x_{fc}\right)=BN\left(f_{fc}\left(\beta \left(f_{conv}\left(f_{mamba}\left(x_{fc}\right)\right)\right)\right)\right), \end{array}$$ (3)

where *f*_*conv*_ is the 1D convolutions with kernel size of 3. β is the Leaky ReLU activation function for regulization. *BN* is the Batch Normalization. *f*_*fc*_ in GLBs are used to either expand the features to twice their original size or compress them to half.

Shallow and deep feature aggregation: we use four GLBs to extract features from student information. Specifically, the first two GLBs utilize fully connected layers to expand the features to twice their original size, while the last two compress them to half. We use the residual connection to link shallow and deep features with the same dimensionality, forming a U-shaped structure, which can mitigate the vanishing gradient problem, aiding in the robust training of the network and enabling the extraction of deeper features. Finally, the resulting features are used for students' depression prediction, which can be expressed as:


$$\begin{array}{c} fx_{glb}^{1}=f_{glb}^{up}\left(x_{fc}\right), \end{array}$$ (4)

$$\begin{array}{c} y=f_{glb}^{dowm}\left(f_{glb}^{dowm}\left(f_{glb}^{up}\left(x_{glb}^{1}\right)\right)+x_{glb}^{1}\right), \end{array}$$ (5)

where $f_{glb}^{up}$ represents the fully connected layer used to expand the features to twice their size, while $f_{glb}^{down}$ represents the fully connected layer used to compress the features to half of their original size.

#### 2.3.2 Factor contribution analysis in GLNet with SHAP

The interpretability of Deep Neural Networks (DNN) has been a challenge for decades (Xu and Yang, 2025; Shah and Sureja, 2025). By explaining the impact of different factors on DNN's decision-making, we can better understand the factors contributing to the formation of depression, helping educators provide timely support for students. Shapley Additive Explanations (SHAP) is a unified method for interpreting deep learning models, which explains the model's output by calculating the SHAP values for each feature. The SHAP value represents the contribution of that feature to the model's output (Lundberg and Lee, 2017). Specifically, the SHAP method calculates the impact of each feature on the final prediction by comparing the model's prediction when the feature is included and excluded, and sums the contributions of all features, which can be expressed as:


$$\begin{array}{c} \phi_{i}\left(f\right)=\underset{S\subseteq N\backslash \left\{i\right\}}{\sum }\frac{|S|!\left(|N|-|S|-1\right)!}{|N|!}\left[f\left(S\cup \left\{i\right\}\right)-f\left(S\right)\right] \end{array}$$ (6)

where *S* is a subset chosen from the feature set *N*, and does not contain the feature *i*. *N* is the full set of features. *f*_*S*_ represents the prediction made by the model trained on the feature set *S*, while *f*(*S*∪{*i*}) is the prediction made by the model trained on the feature set *S* augmented with feature *i*. |*S*| is the size of the subset *S*. |*N*| is the size of the full feature set *N*. The term $\frac{|S|!\left(|N|-|S|-1\right)!}{|N|!}$ is the weight coefficient, which represents the probability of the feature order, reflecting the impact of the position of feature *i* in all possible permutations on the contribution value. We visualize the factor contribution using SHAP values to clearly show the positive and negative contribution of the factors on depression prediction.

### 2.4 Compared methods and evaluation metrics

To validate the effectiveness of GLNet, we compare it with five machine learning methods, including Logistic Regression (LR; Hong et al., 2025), Random Forest (RF; Gohari et al., 2024), XGBoost (Vu et al., 2025), Support Vector Machine (SVM; Risqi et al., 2025), and LightGBM (Song et al., 2025), as well as three deep learning methods, (Baek and Chung, 2020), (Zhou et al., 2024), and DeprMVM (Saha et al., 2024). To ensure a fair comparison, we follow the configurations described in their papers and the public code. Specifically, we set the maximum number of iterations for LR to 100. For SVM, we set the degree of the polynomial kernel to 3. For RF and XGBoost, we use 100 trees for modeling the factors. LightGBM uses Gradient Boosting Decision Trees to model factors for predicting depression. Baek and Chung use dense layers to extract features for predicting depression. Zhou et al. integrated random forest and fully connected layers for depression prediction. DeprMVM combined the MLP and SVM for depression prediction (Baek and Chung, 2020), Zhou et al., 2024).

To evaluate the performance of the models in predicting student depression, we use metrics such as Area Under the Curve (AUC), Accuracy (ACC), Sensitivity (SEN), Specificity (SPE), and F1_Score to quantitatively assess the model's prediction of whether students are in depression.


$$\begin{array}{c} ACC=\frac{TP+TN}{TP+TN+FP+FN}, \end{array}$$ (7)

$$\begin{array}{c} SEN=\frac{TP}{TP+FN}, \end{array}$$ (8)

$$\begin{array}{c} SPE=\frac{TN}{TN+FP}, \end{array}$$ (9)

$$\begin{array}{c} F1\text{\_}Score=2\times \frac{TP}{2TP+FP+FN}, \end{array}$$ (10)

where True Positives (TP) are the number of students correctly predicted as depressed by the model. True Negatives (TN) are the number of students correctly predicted as non-depressed by the model. False Positives (FP) are the number of students incorrectly predicted as depressed by the model, and False Negatives (FN) are the number of students incorrectly predicted as non-depressed by the model. Additionally, we also use a 95% confidence interval to assess the robustness of the models.

### 2.5 Implementation details

We train all models using NVIDIA 2080 Ti GPUs with PyTorch. We standardize all factors to have a mean of 0 and a standard deviation of 1. During training, we use dropout to randomly drop 10% of the factors and apply a 10% data shift for data augmentation to enhance the robustness of the model training. We use the Ranger optimizer (Wright and Demeure, 2021) with an initial learning rate of 1e-4, which is gradually reduced during the training process. We use the weighted cross-entropy loss function to optimize this depression prediction task. The batch size for training is set to 256.

## 3 Results

### 3.1 Comparison with other methods

To ensure the feasibility of contribution analysis using GLNet and its effectiveness in predicting student depression, we compare it with other methods. As shown in Table 2, GLNet achieves the best performance. Specifically, compared to the machine learning method SVM, GLNet improves AUC and ACC by 4.28 and 6.73%, respectively, which may be because GLNet is able to explore deeper information within the data. Compared to the deep learning method DeprMVM, GLNet improves AUC and ACC by 2.89 and 4.04%, respectively, which may be because GLNet effectively explore both global and local information in the data through the Mamba and convolution, demonstrating its effectiveness in predicting student depression. Therefore, GLNet can be used to accurately analyze the impact of different factors on students' depression.

**Table 2:** Comparison of different methods.

**Method**	**AUC(%) (95%CI)**	**ACC(%) (95%CI)**	**SPE(%) (95%CI)**	**SEN(%) (95%CI)**	**F1-Score(%) (95%CI)**
LR	73.11 (72.17–74.18)	73.98 (73.05–74.98)	68.05 (66.38–69.65)	78.18 (76.91–79.41)	77.87 (76.92–78.80)
RF	73.81 (72.80–74.82)	74.57 (73.57–75.57)	69.35 (67.73–71.04)	78.27 (77.10–79.47)	78.28 (77.35–79.18)
SVM	89.08 (88.36–89.85)	82.11 (81.33–83.03)	79.68 (78.40–81.16)	83.84 (82.78–84.90)	84.59 (83.83–85.42)
XGBoost	88.41 (87.73–89.10)	83.24 (82.44–84.10)	79.29 (78.02–80.66)	86.04 (85.07–87.04)	85.74 (85.02–86.51)
LightGBM	72.66 (71.69–73.70)	73.49 (72.56–74.48)	67.75 (66.26–69.53)	77.56 (76.31–78.76)	77.41 (76.41–78.38)
Baek et al.	77.71 (76.78–78.64)	78.37 (77.46–79.27)	73.84 (72.33–75.27)	81.57 (80.45–82.65)	81.54 (80.65–82.40)
Zhou et al.	88.29 (87.65–88.95)	83.69 (82.85–84.52)	83.96 (82.74–85.16)	83.50 (82.42–84.55)	85.71 (84.90–86.47)
DeprMVM	90.47 (89.95–90.94)	84.80 (84.03–85.61)	85.39 (84.19–86.51)	84.39 (83.33–85.42)	86.67 (85.90–87.39)
GLNet	93.36 (93.00–93.68)	88.84 (88.08–89.53)	90.83 (89.80–91.78)	87.42 (86.48–88.40)	90.17 (89.48–90.81)

### 3.2 Effectiveness of weighted cross-entropy loss

To verify the effectiveness of the weighted cross-entropy loss, we compare GLNet with the model without using the weighted cross-entropy loss. As shown in Table 3, the AUC and ACC of the model without using the weighted cross-entropy loss decrease by 2.71 and 2.41%, respectively, demonstrating the effectiveness of the weighted cross-entropy loss.

**Table 3:** Effectiveness of weighted cross-entropy loss.

**Method**	**AUC(%) (95%CI)**	**ACC(%) (95%CI)**	**SPE(%) (95%CI)**	**SEN(%) (95%CI)**	**F1-Score(%) (95%CI)**
w/o WCL	90.65 (88.73–92.85)	86.43 (85.68–87.24)	87.55 (86.50–88.68)	85.64 (84.62–86.65)	88.08 (87.36–88.82)
WCL	93.36 (93.00–93.68)	88.84 (88.08–89.53)	90.83 (89.80–91.78)	87.42 (86.48–88.40)	90.17 (89.48–90.81)

### 3.3 Analysis of factors influencing students' depression

**Figure 2:**
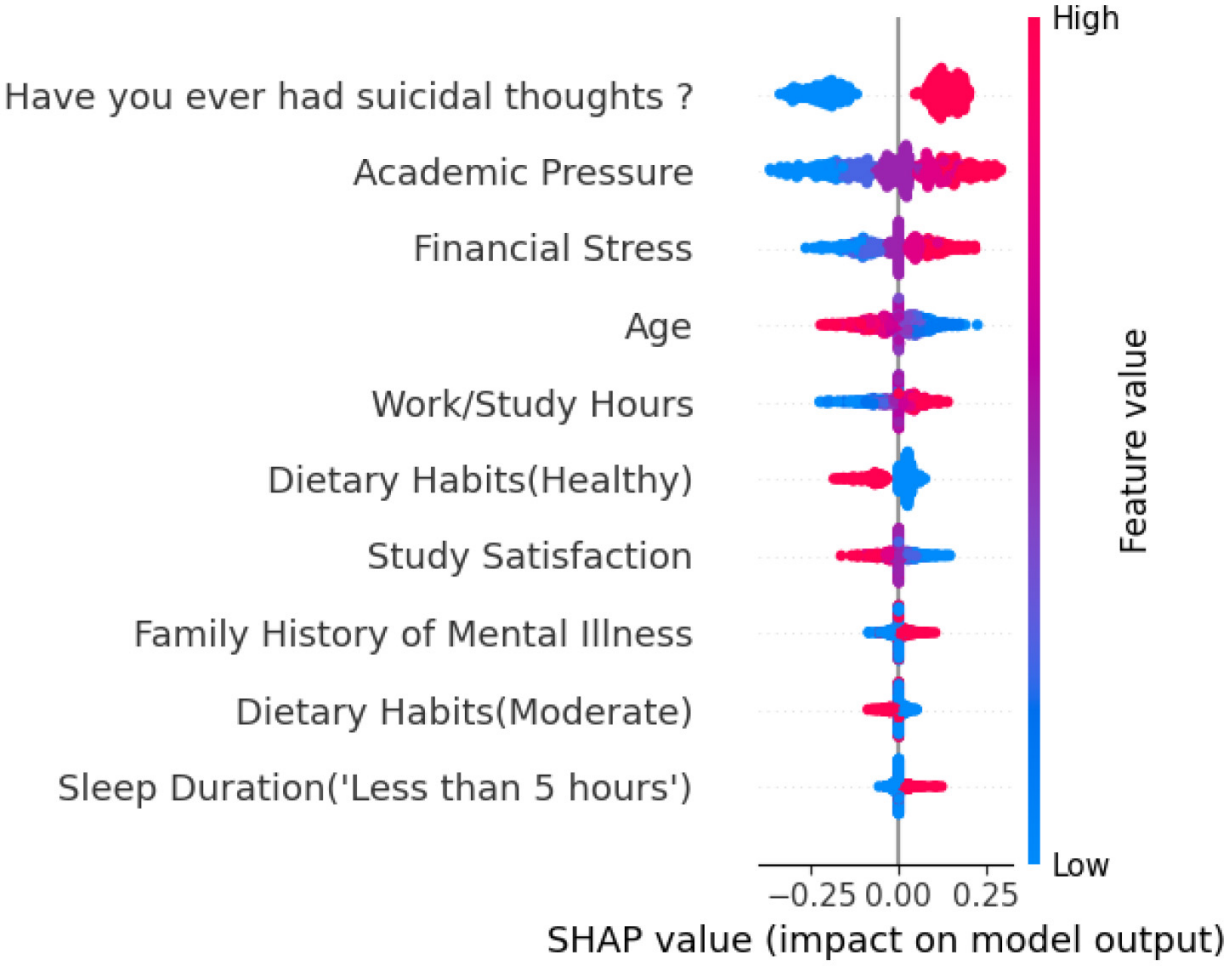
Summary of contributions from different factors on the model's decision. The vertical axis represents the importance ranking of features across different factors, with higher positions indicating greater contribution to the model's decision-making. The horizontal axis denotes SHAP values: the higher the positive value, the greater the contribution to the model's determination of students' depression; conversely, the larger the absolute value of the negative value, the stronger the inhibitory effect on this determination. Each point in the figure corresponds to a student, with the colors reflecting the values of the factors from low (blue) to high (red).

We use the SHAP method (Lundberg and Lee, 2017) to quantify the impact of students' academic and lifestyle habits on their depression. As shown in Figure 2, the *x*-axis represents SHAP values, and the *y*-axis arranges factors based on their impact. Each point represents a sample. We find that having suicidal thoughts, academic pressure, financial stress, work/study hours, family history of mental illness, and sleep duration of less than 5 h are the most significant factors contributing to student depression. In contrast, older age, healthy eating habits, and satisfaction with one's academic performance are typically associated with a lower likelihood of depression. These findings may be helpful for enhancing the optimization of educational healthcare systems.

### 3.4 Analysis of GLNet on data subgrouped by gender

**Figure 3:**
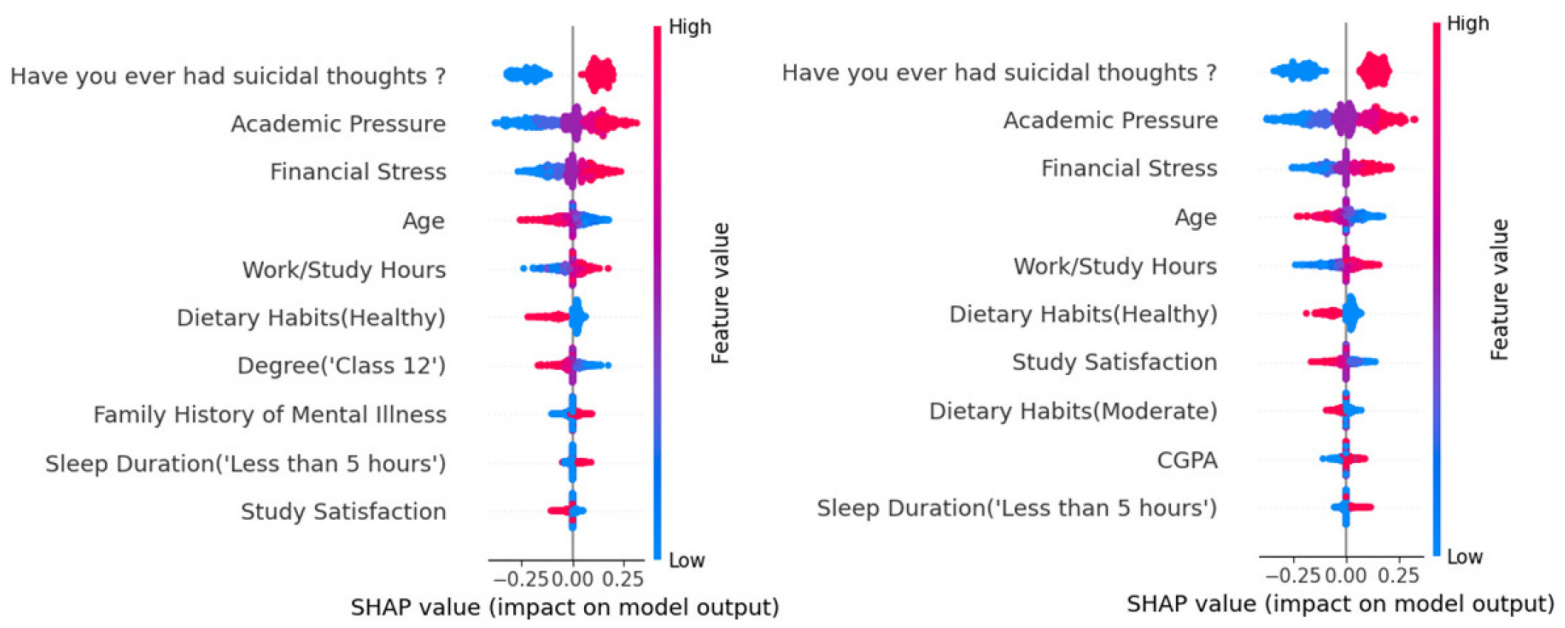
Subgroup analysis of GLNet on students subgrouped by the genders.

To explore the impact of students' academic and life factors on depression across different genders, we conduct a contribution analysis on students of different genders. As shown in Figure 3, we find that for male students, those with an educational level at Grade 12 (senior year of high school) have a lower likelihood of depression. For female students, the higher their Cumulative Grade Point Average (CGPA), the more likely they are to experience depression. These findings may be helpful for enhancing the optimization of educational healthcare systems.

### 3.5 Analysis of GLNet on data subgrouped by educational levels

**Figure 4:**
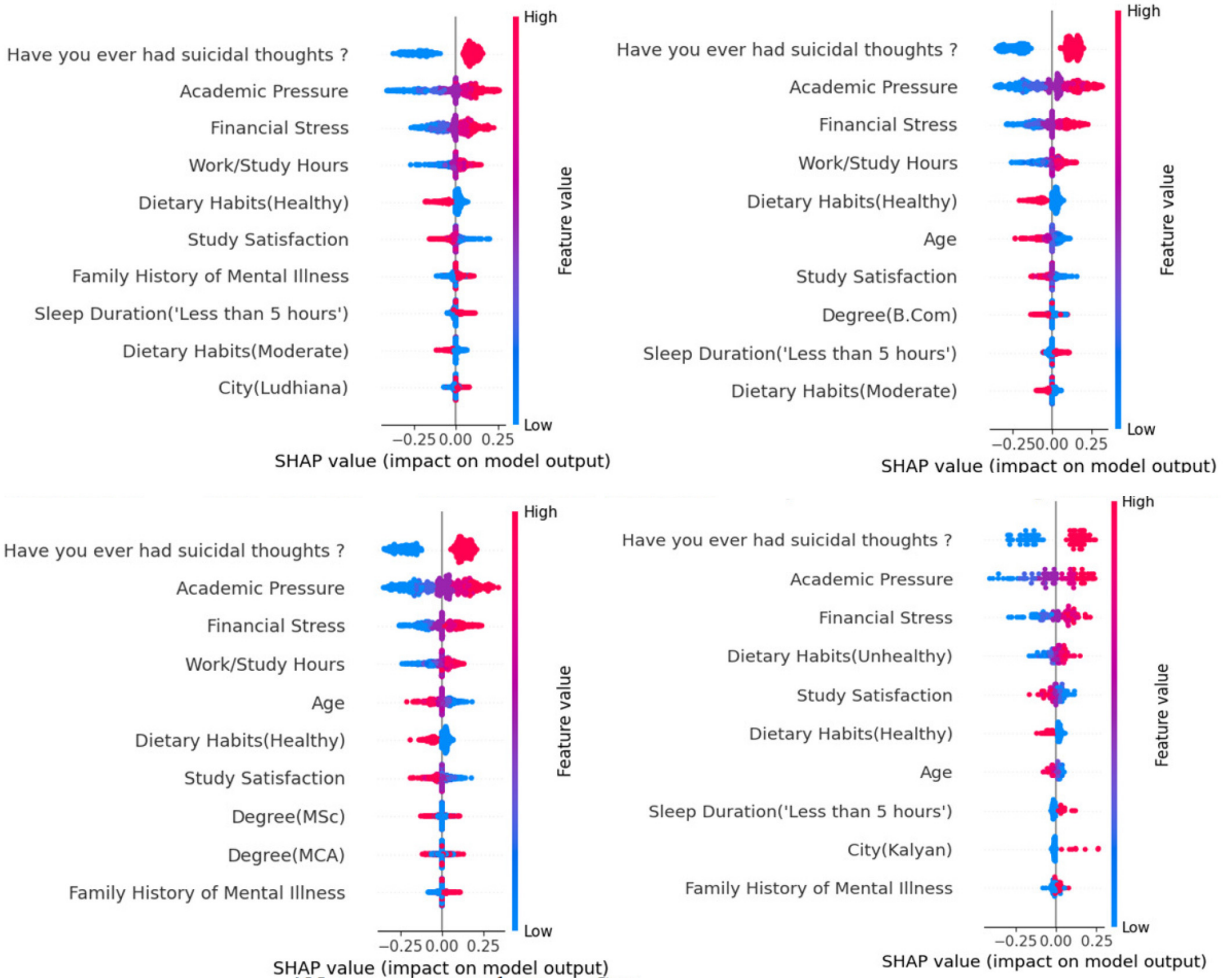
Subgroup analysis of GLNet on data subgrouped by the educational levels.

To explore the impact of various factors on depression among students at different educational levels, we conduct a subgroup analysis on high school students, undergraduates, master's students, and doctoral students. As shown in Figure 4, we find that the effects of different factors on depression vary significantly across students at different educational levels. Specifically, longer working and study hours significantly increase the risk of depression among high school students, undergraduates, and master's students, but have no significant impact on doctoral students. In addition, unhealthy dietary habits significantly increase the risk of depression among doctoral students. These findings may be valuable for enhancing the optimization of educational healthcare systems.

## 4 Discussion

We propose GLNet to explain the impact of various factors of students, such as their demographic, academic, lifestyle, wellbeing, and additional factors, on their depression. GLNet achieves an AUC of 93.36% and an ACC of 88.84% on the publicly available SDD dataset, outperforming other machine learning and deep learning methods, which may be because GLNet effectively explores the global and local information in the factors. In addition, GLNet only takes 0.0006 s to diagnose the depression of a single student, demonstrating its potential value in the application of educational healthcare systems. To our knowledge, we are the first to explore the potential of the latest artificial intelligence framework, Mamba, in predicting student depression and to accurately investigate the predictive factors of student depression. This study quantifies the decision-making process of GLNet using the SHAP method to visualize the potential impact of various factors on student depression.

We find that academic pressure may be associated with student depression. Students often equate academic performance with their own (Park et al., 2007; Fahd et al., 2021; Fairlamb, 2022). If excessive pressure leads to failure to meet expectations, it may trigger low self-esteem, making them feel useless (Nguyen et al., 2019), thereby inducing depression. We also find that students with financial stress may be associated with depression, because students facing a shortage of tuition fees and living expenses constantly worry about whether they can complete their studies and maintain a basic standard of living, and this uncertainty consumes their psychological energy, and gradually turns into depression (Zhang C. et al., 2023). Moreover, comparing themselves to classmates in better financial circumstances may be associated with feelings of inferiority, reducing students' sense of self-worth (Amani et al., 2024). Students who engage in prolonged overstudy may also be associated with depression, as prolonged overstudy leads to neurotransmitter imbalance and induces chronic fatigue (Vieriu and Hainagiu, 2025), weakening the body's tolerance to stress. Moreover, numerous studies confirm that depression has a genetic predisposition (Dionisio-García et al., 2023). Individuals with a family history of mental illness may carry susceptibility genes related to emotional regulation (Liu et al., 2021), making it difficult to regulate negative emotions. In addition, families with a history of mental illness may have unhealthy interaction patterns, such as negative stress-coping styles like excessive criticism and emotional neglect (Reupert and Maybery, 2016; Mitchell and Abraham, 2018; Zhou et al., 2023), and students may acquire these patterns during their growth, increasing their risk of depression. Insufficient sleep also easily contributes to student depression, because insufficient sleep overactivates the emotional centers in students' brains, such as the amygdala, while weakening the rational regulatory centers (Eum and Si, 2025), making students more reactive to negative emotions. Furthermore, we find that suicidal ideation may be associated with depression among students. Although suicidal ideation is one of the core symptoms of depression, it can serve as a strong predictive signal for the occurrence or exacerbation of depression (Tamsir et al., 2025; Wani et al., 2022; Amanat et al., 2022), enhancing the predictive performance of the machine learning model.

Through subgroup analysis, we identify differences in the potential impact of various factors on depression among students of different genders. For male students, those with an educational level at Grade 12 may have a lower likelihood of depression. This may be because Grade 12 is a critical transitional period for further education, with heavy academic tasks but clear goals at this stage (Strom et al., 2022; Fernandes et al., 2023), such as preparing for entrance exams, which prevents them from becoming confused about the future and thus may reduce the likelihood of depressive emotions. For female students, a high CGPA may be associated with their depression. This paradoxical finding offers a compelling real-world illustration of how social psychological theories, such as Social Role Theory and the concept of maladaptive perfectionism, operate in an educational context. This may be due to the dual social and cultural expectations placed on women, as they are required to excel academically while implicitly expected to conform to traditional roles, such as being gentle, family-oriented, and socially adept (Wong, 2007; Horta and Tang, 2023). Female students with high CGPAs may fall into a perfectionist trap due to their overpursuit of “all-round excellence.” As they not only need to maintain outstanding academic performance but may also be expected to behave appropriately in interpersonal relationships, these dual pressures drive them to set excessively high standards for themselves, and once they fail to meet expectations in a given area, they are prone to intense self-criticism, which may be associated with depression.

Through subgroup analysis, we find that different factors may have potential effects on students with different educational levels. Longer working and study hours may be associated with depression among high school students, undergraduates, and master's students, but may have no significant impact on doctoral students. This may be because doctoral students' research is mostly based on independent topic selection, and their long working hours are often associated with intrinsic motivations such as exploring areas of interest and pursuing academic breakthroughs, rather than passively completing standardized tasks (Satinsky et al., 2021). This state of investing in one's own goals can reduce the sense of oppression (Reese et al., 2015; Chazan et al., 2022; Guan et al., 2023). Furthermore, after undergoing academic training at the undergraduate and master's levels, doctoral students have developed more mature stress-coping patterns, and they may be more receptive to the academic norm of “long-term investment and delayed rewards.” In addition, unhealthy dietary habits may be associated with depression among doctoral students. This may be because, compared with students at other academic stages, doctoral students' research work requires more intensive mental labor. Meanwhile, if doctoral students maintain unhealthy diets over the long term, such as insufficient protein intake leading to amino acid deficiency, impairing serotonin synthesis, it can weaken their brain's emotional regulation and fatigue recovery capacities. The combination of this impairment of physiological foundations and academic pressure may make them more prone to developing depression. Furthermore, doctoral students have greater dietary autonomy than students at other academic levels, and long-term unhealthy dietary habits often reflect a reduced priority on self-care (Bayes et al., 2022).

Regarding the potential association between high CGPA and depression among female students, we recommend that educators optimize the existing academic evaluation system to reduce reliance on a single GPA-oriented model and incorporate process-oriented assessments, such as research practices and competency demonstrations. Concurrently, educators should offer counseling programs on perfectionism and psychological adjustment to help female students establish reasonable self-expectations and reduce excessive self-criticism. For doctoral students specifically, we suggest that educators organize thematic lectures on the adaptation of a scientific diet to mental work, provide nutritional guidelines in campus canteens, such as recommendations for high-protein diets, and strengthen doctoral students' self-care awareness to mitigate the potential for depression.

## 5 Limitation and future work

Although this study has made certain progress, there are still some limitations. Firstly, this study mainly explores the impact of students' demographic, academic, and life information on their depression, while other information, such as their interactions with supervisors or classmates, has not been taken into account. In addition, GLNet has only been validated on the SDD dataset collected from cities in India, and its generalizability to other datasets has not yet been verified. In the future, we plan to collect more data to validate the robustness of the model, promoting the potential development of healthcare systems.

## 6 Conclusion

This study proposes a deep learning network called GLNet, which combines global and local information from students' academic and life factors to accurately diagnose student depression. The study finds that high academic and financial pressure, long study and work hours, and insufficient sleep may be associated with student depression. Furthermore, subgroup analyses by gender and education level reveal that female students with high CGPA may be associated with depression, and that unhealthy dietary habits may be associated with depression among doctoral students. These findings provide important empirical support for the prevention and intervention of student depression.

## Data Availability

The original contributions presented in the study are included in the article/supplementary material, further inquiries can be directed to the corresponding authors.
